# Advancing micro-scale cooling by utilizing liquid-liquid phase separation

**DOI:** 10.1038/s41598-018-30584-6

**Published:** 2018-08-14

**Authors:** Wei Xing, Amos Ullmann, Neima Brauner, Joel Plawsky, Yoav Peles

**Affiliations:** 1 Department of Mechanical, Aerospace and Nuclear Engineering, Rensselaer Polytechnic Institute, 110 8th Street, Troy, NY 12180 USA; 2 School of Mechanical Engineering, Faculty of Engineering, Tel-Aviv University, Tel-Aviv, Israel; 3 Howard P. Isermann Department of Chemical and Biological Engineering, Rensselaer Polytechnic Institute, 110 8th Street, Troy, NY 12180 USA; 4 Department of Mechanical and Aerospace Engineering, University of Central Florida, 12760 Pegasus Blvd, Orlando, FL 32816 USA

## Abstract

Achieving effective cooling within limited space is one of the key challenges for miniaturized product design. State-of-the-art micro-scale cooling enhancement techniques incorporate flow disturbances and boiling to reach high performance. However, these methods face the inherent issues of extra pressure drop, flow instability and dry-out that limits heat flux. Here we demonstrate that substantial cooling capability enhancement, up to 2.5 times, is realized by introducing the phase separation of a triethylamine (TEA)/water mixture at the micro-scale. Our experiments show that the enhancement behavior is closely related to the system’s initial composition, temperature, and flow conditions. Moreover, the mixture system exhibits reduced pressure drop after separation, which makes it more promising in serving practical applications. The results reveal new possibilities for liquid coolant selection and provide the experimental foundation for further research in this area.

## Introduction

Effective thermal management — the ability to control the temperature of a system by leveraging the science of thermodynamics and heat transfer — has been a key technological pursuit of modern society. Decades of extensive research and development aimed at enabling the highly efficient operation of equipment, such as gas turbines, medical devices, and arrays of electronic equipment (e.g., electronic cabinets, computer chips, laser diodes, power amplifiers, batteries, motors etc.). In recent years, a new set of challenges has been posted by the miniaturization of powered machines and the development of high performance semiconductor chips, which require advanced micro-scale cooling methods to ensure operational safety. Traditional convective heat transfer enhancement methods include building extended surfaces for increased solid/liquid contact area, stirring to induce flow mixing, using the latent heat of phase change, and so forth^1–6^. Among these strategies, boiling and condensation two-phase flow heat transfer systems are generally regarded to be most effective. However, phase change heat transfer systems are vulnerable to some inherent issues, such as flow instability and critical heat flux, especially at the micro-scale^7,8^. Despite extensive research, these issues often hinder the wide spread use of phase change processes in practical systems.

The phase separation of a partially miscible liquid system is characterized by the formation and movement of fluid domains^9–12^. In several recent publications, it has been shown that this self-propelled motion acts as a stirring mechanism that positively affects thermal transport^13,14^. Poesio *et al*. pioneered the phase separation heat transfer research by confining the water/toluene system in a closed cell. They observed faster temperature response when phase separation was induced^13^. Molin and Mauri used the one-parameter Margules correlation and numerically modeled phase separation process for a quiescent system. Their results indicated that the Nusselt number increases with the mass transfer Peclet number^14^. For convection systems, enhanced thermal transport was also observed^15,16^.

Most phase separation processes are triggered by reducing the system temperature, and thus, they are suitable for heating applications. However, when strong polar interactions are present (such as hydrogen bonding), some liquid mixtures are only miscible below certain temperatures, termed lower critical solution temperatures (LCSTs). An LCST system undergoes phase separation when the temperature exceeds a certain threshold value, and is potentially suitable for cooling applications (See Supplementary Note 1 for thermodynamic point of view of solution behavior). Ullmann *et al*. pioneered the study for phase separation convection cooling with an LCST system, and successfully demonstrated enhanced heat transfer performance^17^. Xing *et al*. first introduced phase separation cooling at micro-scale, and provided experimental evidence for improved heat transfer coefficient^18^. Based on the existing studies, more work needs to be conducted to assist more in-depth understanding of the micro-scale phase separation heat transfer, i.e. geometrical effects, concentration effects etc.

Herein we employ the triethylamine (TEA)/water system, which has an LCST of 18 °C and critical composition of 32.1% TEA mass fraction, to examine its phase separation heat transfer characteristics and multi-phase flow behavior at the micro-scale (See Supplementary Note 2 for detailed information of the binodal behavior of TEA/water mixture; the system’s physical/thermal properties are shown in Supplementary Fig. 1). Our results experimentally demonstrate the feasibility of using an LCST mixture for micro-scale cooling applications, and the effects of initial composition, inlet temperature and heat flux are studied. We find that, at the micro-scale, phase separation at the critical TEA/water composition can enhance heat transfer up to 2.5 times with reduced pressure drop. Two new parameters, an equivalent mass quality, *x*_*w*_, and an augmentation factor, *AF*, were defined to represent the amount of fluid that has separated into two phases and the effectiveness of heat transfer for the phase separating flow over single phase flow. The phase separating flow morphologies, which indicate the concentration field, were observed and characterized against the specific flow conditions. Future research directions and challenges within this subject matter are identified and discussed.

## Results

The convection heat transfer coefficient, *h*, quantifies the effectiveness of a thermal transport process. It measures the amount of heat transferred per unit area per unit temperature difference between the solid surface and the fluid at a given flow condition:

1 $$h=\frac{Q}{A({T}_{w}-{T}_{f})}$$

Here, we apply *h* to the phase separating flow. The calculation formulas of *h* are slightly different, depending on the specific experimental conditions.

### Average thermal transport at the critical composition

**Figure 1:**
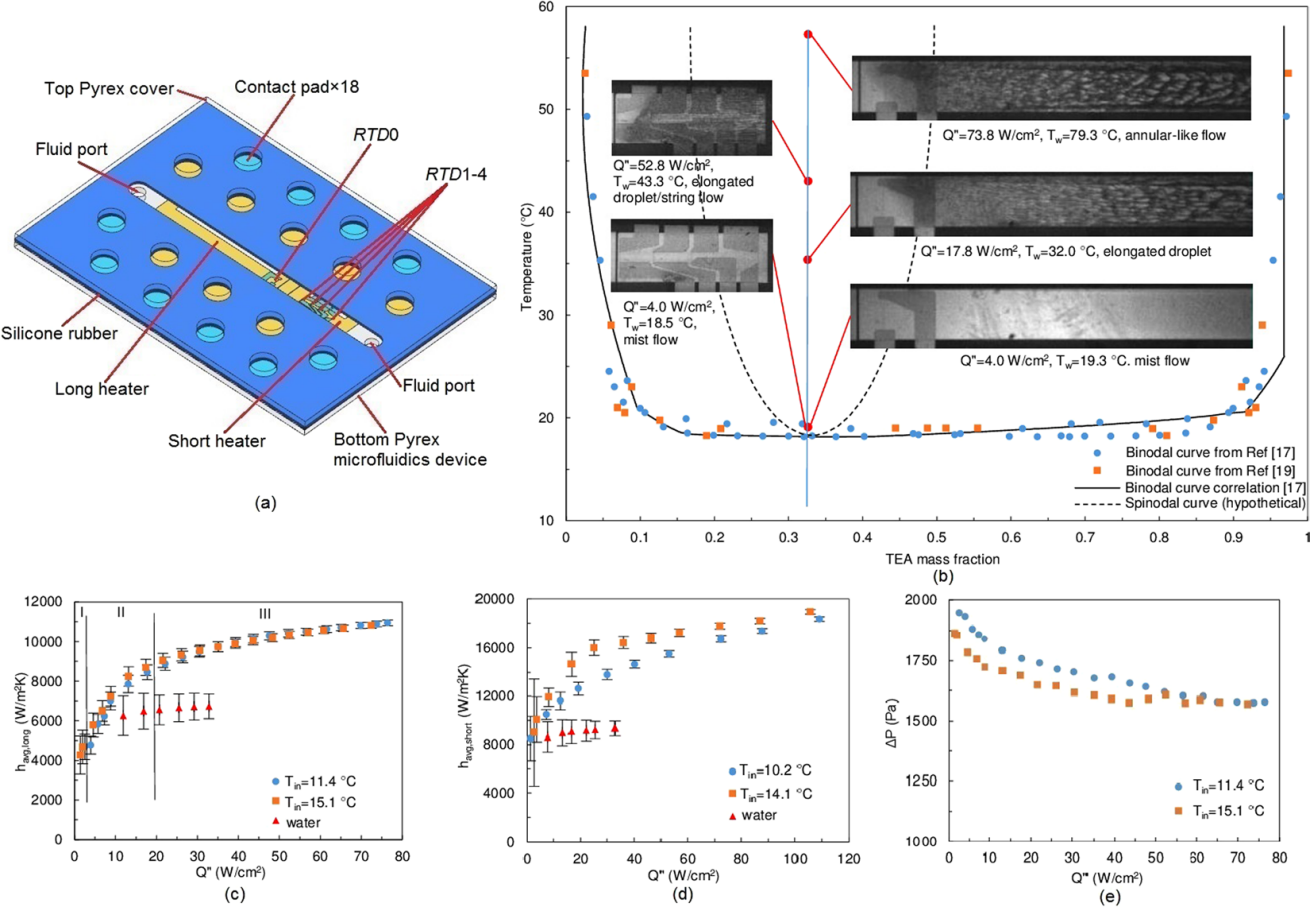
Flow visualization and heat transfer results of critical composition experiments. (**a**) Schematics of the three-layer testing environment. (**b**) Phase diagram and flow visualization images. Photos on the left column are the short heater visualizations (*m*″ = 284 kg/m^2^s, *T*_*in*_ = 14.1 °C), long heater visualizations are on the right column (*m*″ = 208 kg/m^2^s, *T*_*in*_ = 11.4 °C). The system’s location on the phase diagram is approximated using the average wall temperature. For the long heater case with *Q*″ = 73.8 W/cm^2^, *T*_*w*_ = 79.3 °C, its location is marked at the topmost location on the phase diagram. (**c**) Long heater average heat transfer coefficient results at *m*″ = 208 kg/m^2^s, *m*″_*water*_ = 190 kg/m^2^s. (**d**) Short heater average heat transfer coefficients results at *m*″ = 284 kg/m^2^s, *m*″_*water*_ = 274 kg/m^2^s. (**e**) Pressure drop results for the long heater at *m*″ = 208 kg/m^2^s. Full quality flow visualization images can be found in Supplementary Fig. 7.

The average rate of thermal transport was evaluated using a rectangular microchannel structure. The channel was 22 mm long, 2 mm wide, and 0.4 mm deep. Two resistance heaters of different lengths were deposited at the channel bottom. The long heater was 10 mm long and 1 mm wide, and the short heater was 3 mm long with the same width (Fig. 1(a) and Supplementary Fig. 2). The liquid solution entered the heated channel at its critical composition (i.e., at 32.1% TEA mass fraction) and below its critical temperature. When electrical power was applied to the heater, the mixture’s temperature first rose to the critical value (*T*_*critical*_) while the mixture remained single phase. When phase separation occurred, the mixture absorbed heat to break its hydrogen bonds, and separated into liquid phases of different compositions. The equilibrium concentration of each phase was determined by consulting the phase diagram^17,19^ (Fig. 1(b)). At the critical composition, phase separation occurs via spinodal decomposition.

It can be seen from the long heater flow visualization images (Fig. 1(b)) that the multi-phase flow pattern changed with the applied heat flux. At low heat flux (e.g. *Q*″ = 4 W/cm^2^), the flow exhibited a mist flow pattern at the downstream section of the heater, where the fluid temperature was just above the critical value (*T*_*critical*_). With the increasing heat flux (e.g. *Q*″ = 18 W/cm^2^), the onset of phase separation propagated upstream, and the phase separating flow covered a larger heater area. Since the fluid was heated above *T*_*critical*_, the system was far removed from its initial equilibrium state. The fluid domains are distinguishable with an elongated droplet flow pattern. As the heat flux continued to increase (e.g. *Q*″ = 74 W/cm^2^), the onset of phase separation remained unchanged, but the region of phase separation penetrated deeper into the fluid bulk. The fluid domains are more distinct, and the flow pattern becomes annular-like (note that no phase separation occurred at the top layer of the flow passage). The flow field on the short heater resembles the first 1/3 of the long heater.

Figure 1(c and d) depict the average heat transfer coefficient, *h*_*avg*_, for both the long and the short heaters at fixed mass fluxes (*m*″). *h*_*avg*,*long*_ is characterized by three zones (Fig. 1(c)): Zone I, at a heat flux of *Q*″ ~ 2 W/cm^2^, was a region where the mixture remained single phase; in Zone II, *h*_*avg*,*long*_ increased rapidly with heat flux (*Q*″ ~ 4–18 W/cm^2^); and in Zone III, *h*_*avg*,*long*_ stabilized at perhaps an asymptotic value (*Q*″ > 18 W/cm^2^). Because of the smaller area, the total heat transferred to the fluid from the short heater was much less than that transferred from the long heater when both operated at the same heat flux. Therefore, *h*_*avg,short*_ increased, but the three-zone behavior was less apparent. The flow images provide insight into the three-zone behavior. Zone II corresponds to the expansion of the phase separated region at the heater/liquid interface, and thus, a larger area of the heated solid surface is experiencing the heat transfer benefit caused by phase separation. This leads to a rapid increase of *h*_*avg*,*long*_. Once the phase separation region ceases to propagate upstream and extends into the fluid bulk perpendicular to the heated wall, the enhancement of the thermal transport slows down. Thus, *h*_*avg*,*long*_ increases at a diminishing rate in Zone III. The phase separation flow exhibits greater heat transfer coefficient, compared to the case where water is used as the coolant at a similar mass flux (Fig. 1(c)).

The effect of fluid inlet temperature (*T*_*in*_) is also examined in Fig. 1(c and d). At low heat fluxes, a mixture with higher *T*_*in*_ is closer to the critical temperature, and thus shows higher *h*_*avg*_. However, at intermediate to high heat fluxes, the effect of *T*_*in*_ yields no significant difference in *h*_*avg*_. Due to the shape of the binodal curve, after the onset of phase separation, the compositions of the separated phases are nearly independent of the fluid temperature (Fig. 1(b)). Therefore, the thermal transport performance is nearly independent of the fluid temperature (as well as the heat flux), once the system temperature is much higher than *T*_*critical*_. The viscosities of the separated phases are both less than that of the mixture (Supplementary Fig. 1(b)), and so the pressure drop of the system decreases after phase separation (Fig. 1(e)).

### Local thermal transport at the critical composition

**Figure 2:**
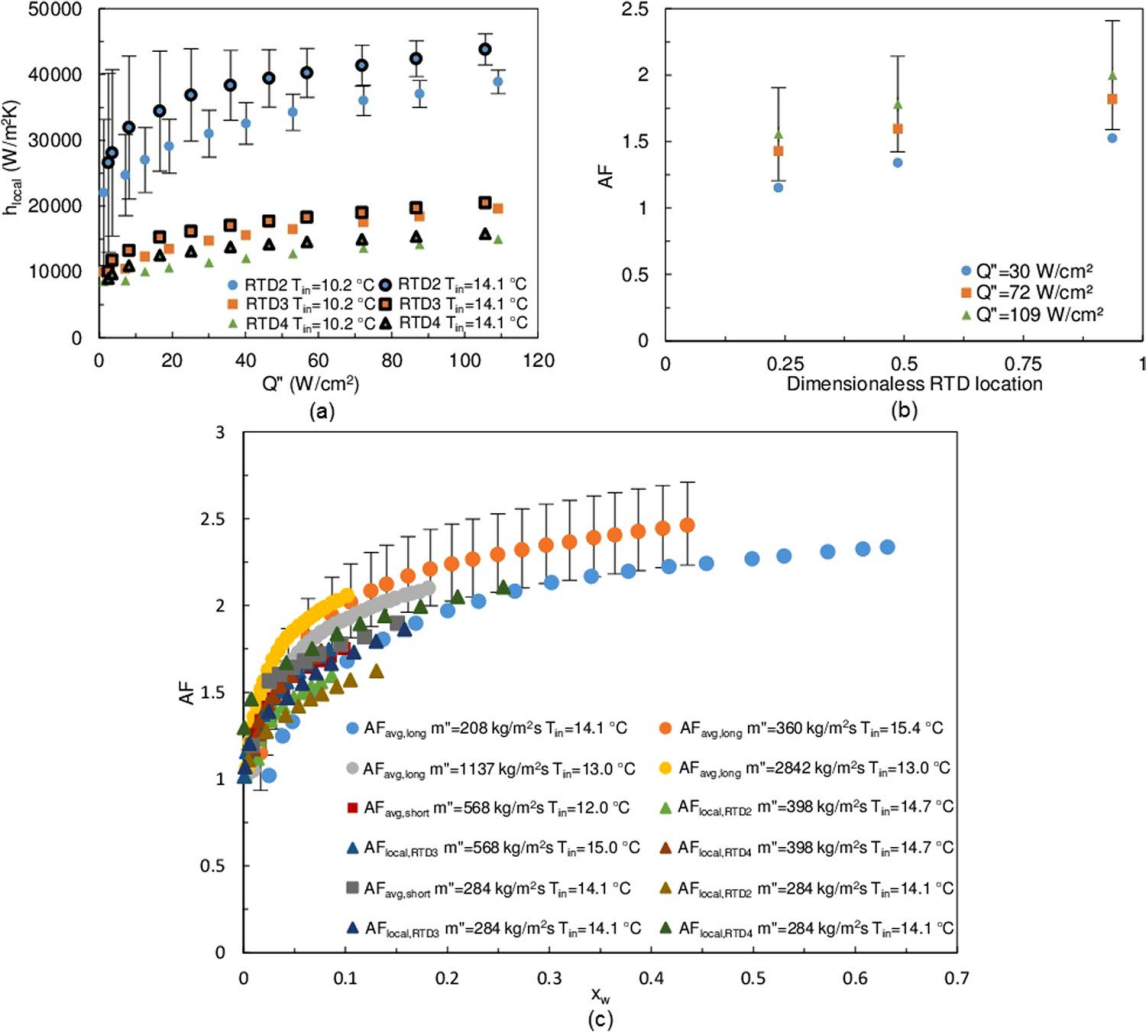
Heat transfer coefficients and *AF* data for the critical composition. (**a**) Local heat transfer coefficients on the short heater, *m*″ = 208 kg/m^2^s. The fluid flows from *RTD*2 to *RTD*4 in local measurement experiments. (**b**) *AF* by locations of *RTD*s, *m*″ = 284 kg/m^2^s, *T*_*in*_ = 10.2 °C. (**c**) *AF* vs. mass quality at various experimental conditions. For clarity, not all uncertainties are plotted.

The critical composition local thermal transport process was examined with localized measurements using resistance temperature detectors (*RTD*s). The *RTD*s were located along the flow direction at several locations (Supplementary Fig. 2). The three-zone behavior is indistinct for *h*_*local*_ (Fig. 2(a)), since the total heat input was less from the short heater. Because *RTD2* locates at a more upstream location than *RTD3* and *RTD4* (Supplementary Fig. 2), the entry length effect results in the greatest local heat transfer coefficient among all *RTD*s.

To further analyze the thermal transport characteristics, the data is presented in a non-dimensional form. Borrowing the concept from flow boiling, an equivalent mass quality, *x*_*w*_, was defined based on the wall temperature. The flow quality presents the percentage of fluid that has separated into two phases assuming that the enthalpy of mixing is dependent upon the wall temperature^20–22^. An augmentation factor, *AF*, was defined as the ratio of *h* between the phase separated flow and the single phase flow of the same mixture at the same flow rate and inlet conditions. Figure 2(b) shows the local *AF* at various locations along the heater. As the mixture moves downstream and receives more energy, *AF*_*local*_ increases due to the enthalpy of phase separation and the flow mixing effect. The maximum *AF*_*local*_ is observed at *RTD*4, where the mass quality peaks. With the aid of the dimensionless parameters, all experimental data at the critical composition can be superimposed onto a single plot (Fig. 2(c)). *AF* and *x*_*w*_ clearly show an asymptotic behavior, and this behavior holds for different heating lengths, inlet temperatures and flow rates at both the average and local levels. The maximum *AF* observed during experiment is about 2.5. The rapid increase for *h*_*avg*_ occurs at mass qualities less than 0.1, indicating that *AF*_*avg*_ is mainly boosted by phase separation in the vicinity of the wall, not in the bulk of the fluid. Similarly, *AF*_*local*_ also experienced a rapid increase in this range of quality. It is proposed that the maximum *AF* occurs when the flow quality reaches unity, indicating that all the mixture undergoes phase separation.

### Thermal transport at off-critical compositions

**Figure 3:**
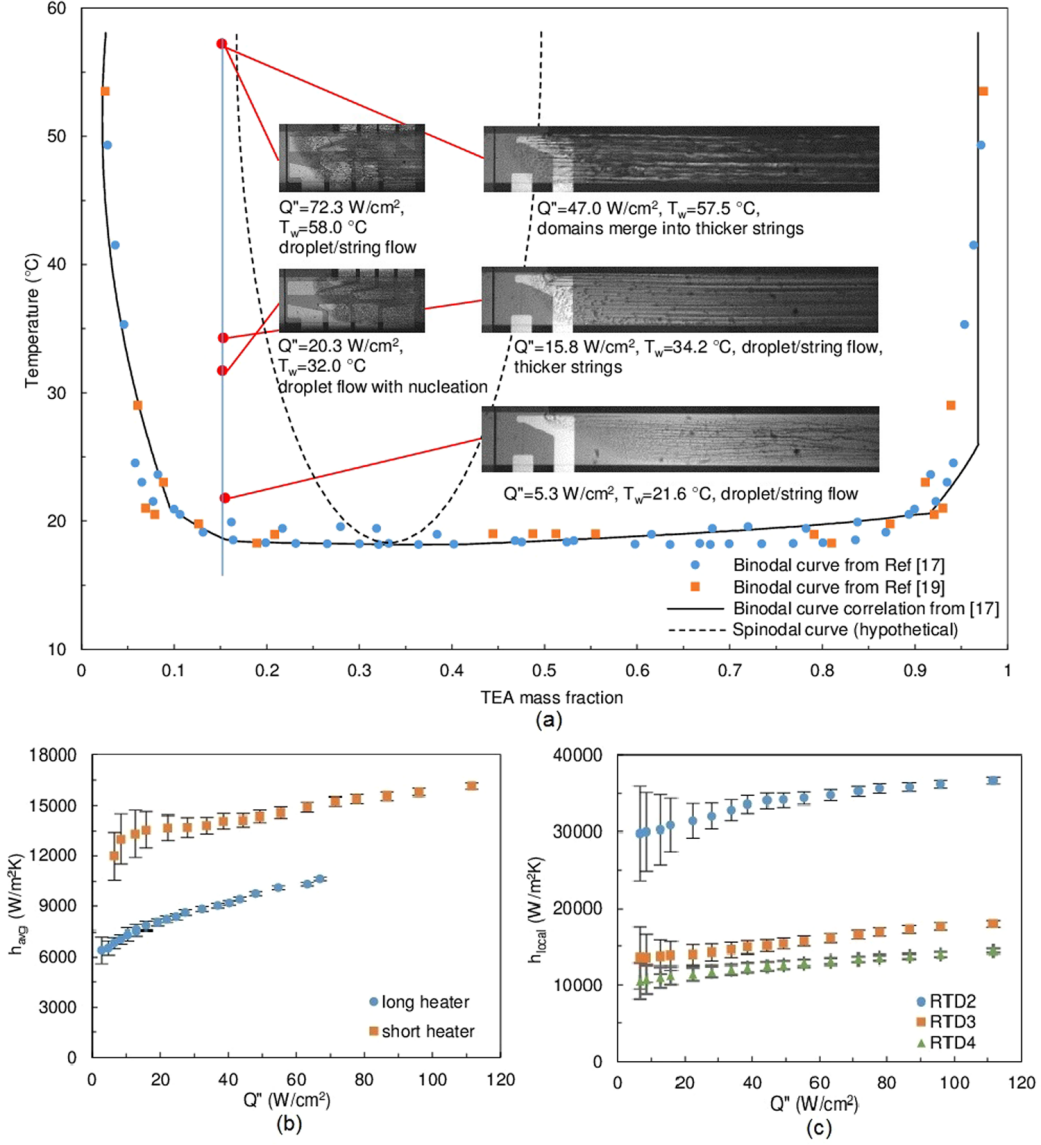
Flow visualization and heat transfer results summary of 15% TEA mass fraction experiments. (**a**) Phase diagram and flow visualization images. Photos on the left column are the short heater visualizations (*m*″ = 215 kg/m^2^s, *T*_*in*_ = 14.6 °C), long heater visualizations are on the right column (*m*″ = 241 kg/m^2^s, *T*_*in*_ = 12.8 °C). The system’s location on the phase diagram is approximated using the average wall temperature. (**b**) Average heat transfer coefficient results at *m*″ = 402 kg/m^2^s, *T*_*in*_ = 12.8 °C. (**c**) Short heater local heat transfer coefficient results at *m*″ = 358 kg/m^2^s, *T*_*in*_ = 14.0 °C. Full quality flow visualization images can be found in Supplementary Fig. 8.

Two off-critical concentrations, 15% and 50% TEA mass fraction, were examined. At off-critical compositions, phase separation proceeds by nucleation and domain growth, which is a localized and less rigorous process, compared to spinodal decomposition^23–25^ (See supplementary Note 3 for more information about nucleation and spinodal decomposition). For the 15% mixture, the phase separation morphology clearly exhibits a nucleation pattern (Fig. 3(a)). As more heat is delivered to the fluid, the string shape regions coalesce to form thicker strings. Under bulk advection and the shear force, the resulting flow pattern resembles a droplet/string flow. Flow visualization on the short heater confirms the results from the long heater and provides a closer look at the drop-like domains. The heat transfer coefficient using a 15% mixture increased almost linearly (Fig. 3(b and c)) with applied heat flux, both on average and on local basis. However, the enhancement ratio was weaker than the case of critical composition.

**Figure 4:**
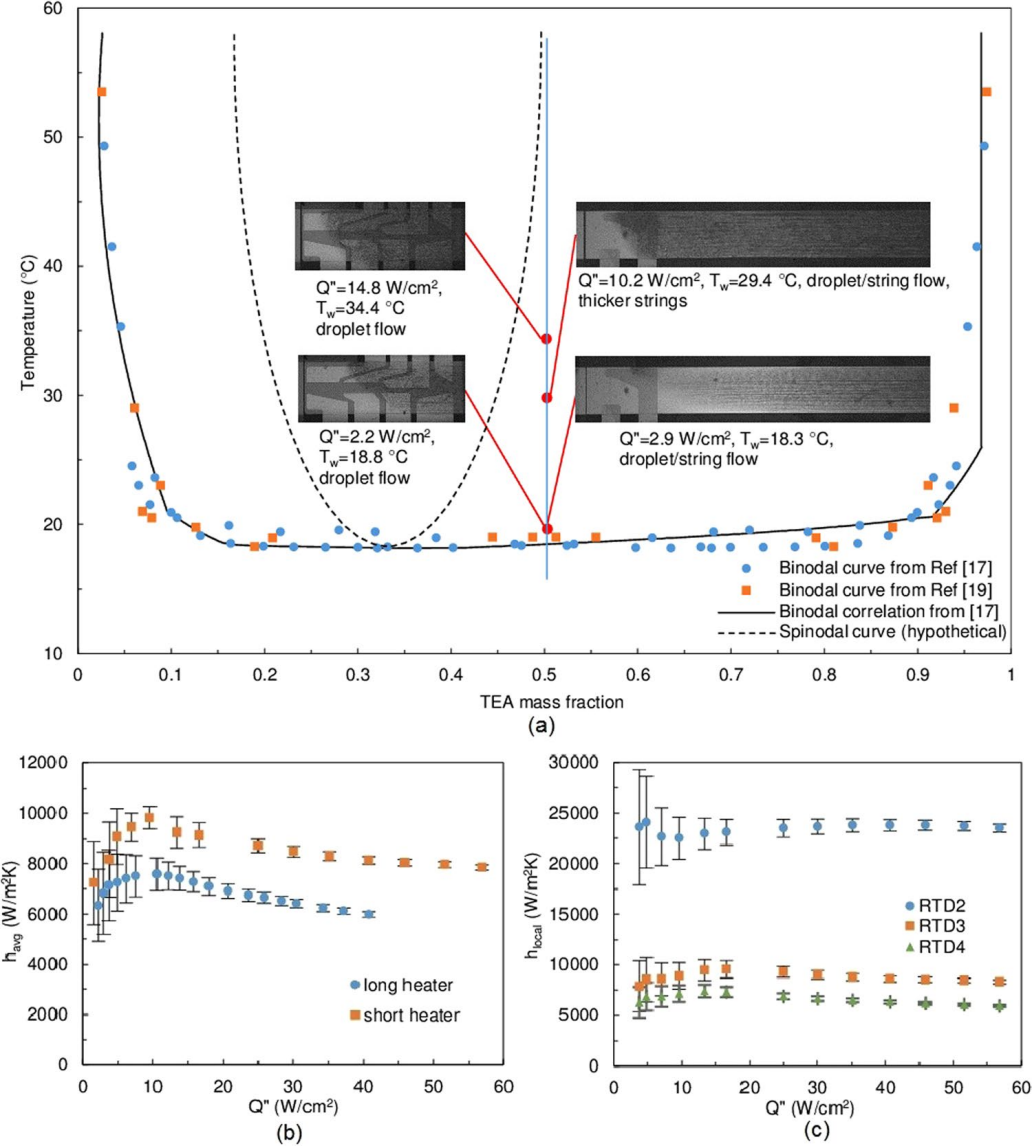
Flow visualization and heat transfer results summary of 50% TEA mass fraction experiments. (**a**) Phase diagram and flow visualization images. Photos on the left column are the short heater visualizations (*m*″ = 358 kg/m^2^s, *T*_*in*_ = 14.5 °C), long heater visualizations are on the right column (*m*″ = 178 kg/m^2^s, *T*_*in*_ = 12.2 °C). The system’s location on the phase diagram is approximated using the average wall temperature. (**b**) Average heat transfer coefficient results at *m*″ = 358 kg/m^2^s, *T*_*in*_ = 11.8 °C. (**c**) Short heater local heat transfer coefficient at *m*″ = 358 kg/m^2^s, *T*_*in*_ = 13.8 °C. Full quality flow visualization images can be found in Supplementary Fig. 9.

Similar to the 15% mixture, the 50% mixture exhibited a droplet/string flow pattern, but the sizes of the droplets and strings were smaller (Fig. 4(a)). At this composition, *h* first increased slightly and then dropped to values below the corresponding single phase flow *h*. According to the lever rule, after phase separation, the 15% mixture has more content of the thermally favorable phase (water-rich) than the 50% mixture (Table 1). As a result, the majority of the solid/liquid interface was covered by the favorable phase for the 15% mixture. On the other hand, the 50% TEA mixture benefits the most from heat of mixing effects (Table 1). With the onset of phase separation, latent heat and flow mixing boost the thermal energy transport. However, the solid/liquid interface was gradually covered by the unfavorable phase (TEA-rich), due to its greater wettability on the device surface (Supplementary Fig. 3). As a result, the heat transfer process from the wall to the bulk of the fluid is hindered, i.e., a layer of low thermal conductivity material inhibits thermal energy transport. Consequently, the overall heat transfer performance deteriorates compared to the corresponding single phase flow at the same mass flux. It is shown that the 50% mixture yields reduced cooling performance, while the 15% mixture shows good heat transfer enhancement. However, the heat transfer coefficient for the 15% mixture varies considerably with input heat flux with no asymptotic behavior, such that *h* is sensitive to heat flux. For practical applications, the actual performance is difficult to predict. As a short conclusion, the critical composition mixture seems to exhibit desirable performance augmentation.

**Table 1:** Comparison of water-rich phase percentage (*x*_*h*_), TEA-rich phase percentage (*x*_*l*_) and enthalpy of mixing between critical composition, 15% and 50% TEA mass fraction, assuming the system’s temperature is 50 °C.

Composition	*x*_*h*_ (water-rich phase)	*x*_*l*_ (TEA-rich phase)	*Δh*_*mix*_ (kJ/kg)
32.1% (critical)	64.8%	35.2%	46.2
15%	86.5%	13.5%	29.5
50%	49.1%	50.9%	50.6

Similar to spinodal decomposition, the nucleated droplets coalesce to form bigger domains. Interestingly, at the downstream section of the heater, where the fluid near the wall reaches higher temperatures, the flow pattern seems to be string flow, which is different from the spinodal decomposition pattern. Generally, as a mixture reaches higher temperatures, it transitions from a nucleation region into a spinodal decomposition region (as shown in the phase diagram). However, this was not observed during our experiments. Two scenarios are possibly involved here. The spinodal curve is narrow, such that spinodal decomposition only occurs at compositions very close to the critical composition. Alternatively, as nucleation occurs in the heater’s upstream section, the mixture immediately separates into different phases. As the separated fluids flow downstream, heat is transferred to the separated phases that are already at concentrations determined by the binodal curve. That is to say, the fluid mixture always stays within the gap between the binodal and spinodal curve.

Observation of the 15% mixture also reveals that the two separated phases (i.e., water-rich and TEA-rich) end up with larger droplets than the 50% mixture. The size of the droplets is determined by the principle of minimizing the Gibb’s free energy of the system. Therefore, the dependence of Gibb’s free energy on the system’s concentration might be different in both cases. The different droplet sizes might also be linked to the difference in the systems’ viscosities (Supplementary Fig. 1(b)) and the hydrodynamics of the flow field. The viscous force tends to tear the fluid domains into smaller droplets, and thus, the 50% mixture (greater viscosity) tends to separate into domains with smaller sizes.

### Flow visualization and image analysis at the critical composition

**Figure 5:**
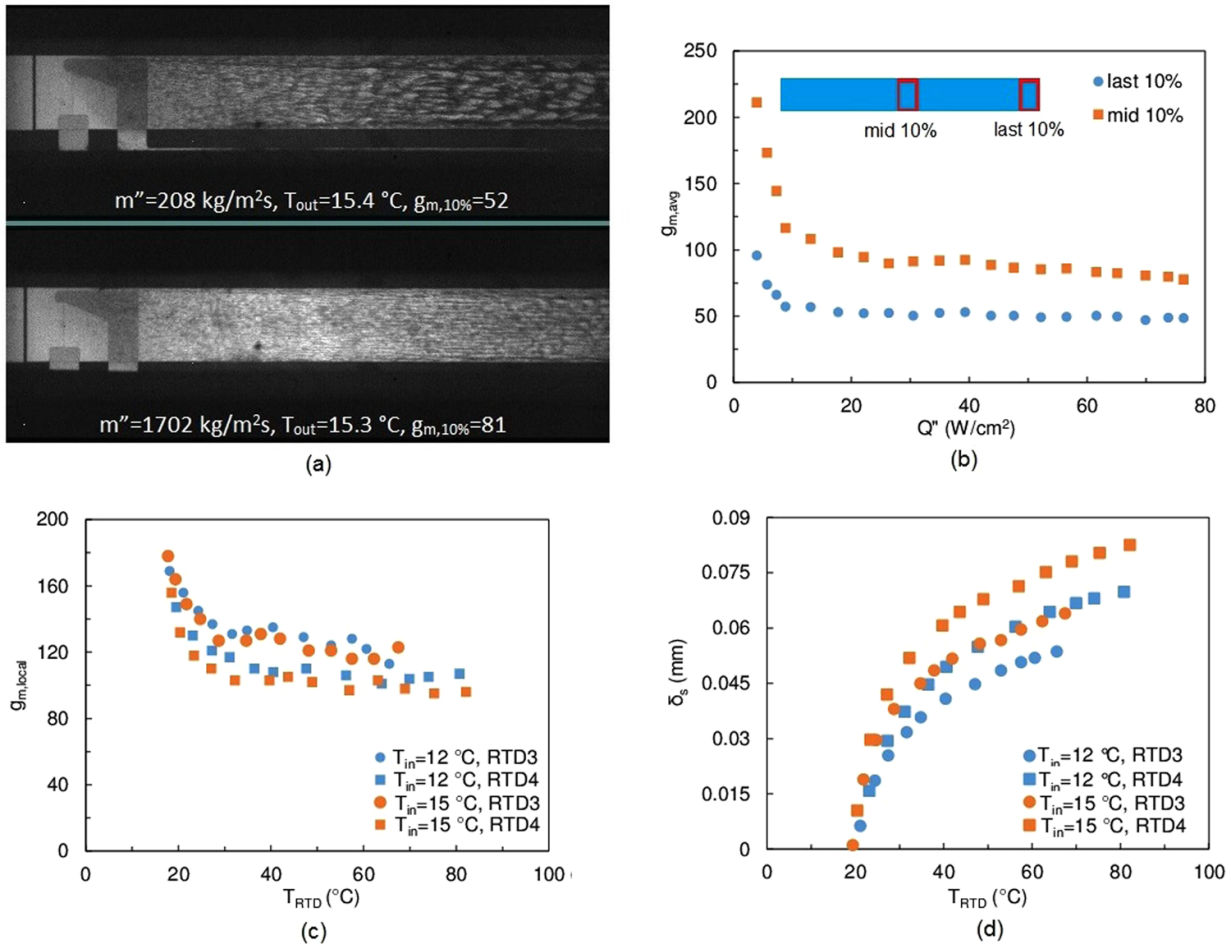
Critical composition flow visualization analysis. (**a**) direct comparison of flow pattern of similar fluid outlet temperatures but different flow rates. *g*_*m,10%*_ is the average gray value of the last 10% length of the long heater. (**b**) average gray value at the middle and last 10% of the long heater at *m*″ = 208 kg/m^2^s, *T*_*in*_ = 11.4 °C. (**c**) *RTD* local gray value at *m*″ = 567 kg/m^2^s. (**d**) separation layer thickness estimation at *m*″ = 567 kg/m^2^s.

For spinodal decomposition of quiescent systems, the morphology of the domains has been shown to be bi-continuous and dendritic^11,26,27^.However, flow visualization in the current study shows a different result: the morphology during spinodal decomposition changes with forced advection^28,29^. At a given flow rate, the overall concentration field evolves with increasing heat flux and the fluid temperature (Fig. 1(b)). For the same average fluid outlet temperature, the flow pattern varies at different flow rates (Fig. 5(a)). It is postulated that the flow morphology also depends on the fluid’s residence time in the heated channel. Because the TEA-rich phase appears darker (greater turbidity) than the single phase mixture and the water-rich phase, a gray value, *g*_*m*_, can serve as an indicator for TEA concentration in the separated phases.

The gray value image analysis technique was first applied to the long heater. The average gray values of the middle 10% and the last 10% length of the heater were calculated (Fig. 5(b)). The sample images were extracted from 20 different frames from the flow visualization videos. The first comparison was made between different heat fluxes under the same flow rate. With increasing heat flux, the mean gray value declined, indicating the presence of darker shades resulting from phase separation. The gray value reached an asymptote at a heat flux of about 18 W/cm^2^. The asymptotic characteristics of *g*_*m*_ is consistent with the trend observed for *h*_*avg*_ (Fig. 1(c)). Figure 5(a) also presents a comparison between different flow rates with similar outlet mean fluid temperature. Higher flow rates correspond to shorter residence times, which leads to a higher mean gray value in the last 10% length of the heater.

The separated phases reside in the sub-layer of the thermal boundary layer where the local temperature exceeds *T*_*critical*_. The thickness of this phase separation boundary layer, denoted as *δ*_*s*_, is linked to the local mass quality and substantially affects the observed gray value. The local gray value (*g*_*m,local*_) analysis was conducted while assuming that the temperature of the fluid close to the heated wall could be approximately measured by the wall *RTD*s. The same gray value acquisition technique was applied at the *RTD* locations. Due to the absence of a non-isothermal phase separation flow model, a single phase conjugate heat transfer model was used to evaluate the separation boundary layer thickness. To match the simulated wall temperatures with measured values, both the mixture specific heat (Supplementary Fig. 1(a)) and an effective thermal conductivity (*k*_*eff*_ = 0.68 W/mK) is used, noting that the value of *k*_*eff*_ is about twice of the thermal conductivity of the single phase mixture, and is greater than that of the water (for details of the model see Supplementary Note 4). This indicates that both the latent heat effect and lateral mixing induced by phase separation are responsible for the heat transfer enhancement.

Figure 5(c) depicts the mean gray value on top of *RTD*3 and *RTD*4 at different fluid inlet temperatures. It can be seen that the inlet temperature has very little effect on *g*_*m,local*_. However, for gray values at *RTD*3 and *RTD*4 at the same inlet temperature and wall temperature, major differences are observed due to different residence times and separation boundary layer thicknesses (Fig. 5(d)). For *RTD*3 with *T*_*in*_ = 15.0 °C and *RTD*4 with *T*_*in*_ = 12.0 °C, *δ*_*s*_ is almost identical. Thus, the difference in residence time accounts for the difference in *g*_*m*,*local*_. However, comparing *g*_*m*,*RTD3*_, *T*_*in*_ = 12.0 °C and *g*_*m*,*RTD4*_, *T*_*in*_ = 15.0 °C, it can be concluded that both the residence time and the separation layer thickness affect *g*_*m*,*local*_. When *T*_*RTD*_ = 40 °C, *g*_*m*,*RTD*_ reaches an asymptotic value, which is in agreement with the shape of the binodal curve.

Another important observation through the flow visualization images (Fig. 5(a)) is that the dark and light shades are not completely separated apart from one another. It is clearly to see that some dark shades are inside of the lighter shade domains. As the flow is three-dimensional and the camera was set to provide a top view, the observation might be due to the overlap of separated domains. Apart from this experiment-induced uncertainty, the complex flow conditions, i.e. presence of bulk advection, sharp temperature gradient, preferable wetting of one component, cause a mismatch in time response between the geometrical coarsening induced by hydrodynamic effect and mass diffusion driven by thermodynamic effect^30–32^.

## Discussion

The current work demonstrates that phase separation of a TEA/water mixture can enhance thermal energy transport at the micro-scale by up to 2.5 times compared to a single phase flow of the same mixture. Significant thermal transport enhancement over water is also observed. At the critical composition, both the local and average heat transfer coefficients of the mixture increase asymptotically with heat flux. Data from the critical composition mixture flow experiments over a range of flow rates, inlet temperatures, heat fluxes, and average and local measurements, suggest a unified heat transfer characteristic. A rapid increase in *AF* occurs at low *x*_*w*_ values (less than 0.1), demonstrating that enhanced heat transfer is mainly the result of phase separation at the vicinity of the wall, not in the fluid bulk. The mass quality analysis implies that the theoretical maximum *AF* is reached when *x*_*w*_ = 1. However, due to the non-uniform temperature distribution in the flow, the maximum *x*_*w*_ cannot be reached. The pressure drop decreases after phase separation due to a reduction in the system’s viscosity, which is an additional benefit. The water-rich (15%) composition shows some heat transfer enhancement, but not as much as the critical composition. The TEA-rich (50%) mixture yields unfavorable heat transfer performance and is not suitable for any cooling applications. Critical composition phase separation flow shows a mist flow, elongated droplet flow, and annular-like flow pattern, while off-critical compositions show a droplet/string flow pattern. It has been shown in the flow visualization analysis that both thermodynamics and hydrodynamics affect the evolution of flow pattern and the concentration field.

The asymptotic thermal transport behavior is mainly caused by the behavior of the binodal curve for the TEA/water system and might vary depending on the mixture’s content. The domain morphology for forced flow deviates from the quiescent case due to the presence of inertial and shear forces. Since the flow is non-isothermal, the temperature distribution determines the concentration field. As the local temperature varies, so do the local compositions of the separated phases. On the other hand, the fluid away from the heated surface might not reach the critical temperature and remain single phase. The resulting flow consists of multiple phases with concentrations determined by the local temperature.

Beside the TEA/water mixture, other possible fluid systems need to be studied. The different thermal/physical properties as well as phase separation characteristics can lead to different thermal transport and multi-phase flow behavior. While studying various fluid mixtures, the mixture’s physical/thermal properties need to be carefully measured. It has been proven that the critical solution temperature can be adjusted by adding miniscule amount of a third component. Therefore, the mixture’s critical temperature can be adjusted to satisfy an application’s specific needs. Once the desirable liquid/liquid mixture is configured, it can be readily applied without substantial modification to the cooling equipment. This makes the application of such partially miscible fluid systems promising. The physics of the process requires deeper understanding, as the time scales of the mass, momentum and thermal transport are interlinked during the process. The transient interactions of the transport phenomenon are of great interest, and more detailed studies are needed. Meanwhile, the liquid/liquid interface deserves more attention as strong chemical potential gradient presents.

## Methods

### Experimental setup for measuring heat transfer coefficient

The TEA/water solution is stored in a 316 Stainless Steel tank, which is immersed in a cooling water bath. A magnetic stir bar constantly mixes the fluid solution, which is maintained below its critical solution temperature. The solution temperature in the tank is monitored by a thermocouple. The fluid is delivered to the test section using a micro pump, and a 60-micron pore size filter is placed between the storage tank and the pump to protect the gear pump from unexpected contaminants. Two pressure transducers are placed at the inlet and outlet of the test section. A flowmeter is placed after the test section where the fluid mixture reaches the ambient temperature (Supplementary Fig. 4). The test section is a rectangular micro channel whose cross section is 2 mm wide and 0.4 mm high. Two resistance heaters of length 10 mm and 3 mm are deposited on the channel bottom, and five *RTD*s are also deposited on the channel bottom. The *RTD* layer is separated from the heater layer by a 1 µm thick layer of silicon dioxide. The bottom Pyrex substrate, one piece of 0.4 mm thick silicone rubber, and the top transparent Pyrex cover are pressed together to form the microchannel. The test section is enclosed in a device package to allow for fluid flow and electrical connections (Supplementary Figs 2 and 4).

### Heat transfer coefficient calculation

The average heat transfer coefficient calculation for critical composition is performed according to:

2 $${h}_{avg}=\frac{Q^{\prime\prime} }{{T}_{w,avg}-{T}_{f}}$$

where *Q*″ is the effective heat flux after subtracting heat loss; *T*_*w,avg*_ is the average wall temperature measured by the heater resistance, note that the higher *T*_*w,avg*_ achieved in experiments at a given flow was about 80 °C; *T*_*f*_ is the average fluid temperature inside the channel, and it is calculated as half of the sum of fluid inlet temperature, *T*_*in*_, and mean fluid outlet temperature, *T*_*out*_. *T*_*in*_ is measured by *RTD*0 assuming the local wall temperature is in equilibrium with the fluid bulk. *T*_*out*_ is calculated via a numerical integration using energy balance and specific heat data of the mixture at critical composition (*ṁ* is the mass flow rate of the mixture), noting that the specific heat data (Supplementary Fig. 1(a)) takes the latent heat into consideration.

3 $$Q=\dot{m}{\int }_{{T}_{in}}^{{T}_{out}}\,{c}_{p}dT$$

The local heat transfer coefficient is calculated according to:

4 $${h}_{local}=\frac{Q^{\prime\prime} }{{T}_{w,local}-{T}_{f,local}}$$

where *T*_*w,local*_ is the *RTD* temperature measurement, *T*_*f,local*_ is the mean fluid temperature at the corresponding *RTD* location, which is calculated by the inlet temperature and energy balance similar to *T*_*out*_. For all off-critical compositions, due to the absence of specific heat data, *T*_*out*_ cannot be calculated. Alternatively, only the fluid inlet temperature is used in the denominators.

### Temperature measurements

Besides the *RTD*s, the heaters are also used as temperature sensors. During experiments, two Digital Multi-Meters (DMMs) measure the current and voltage across the heater, thus the heater resistance is obtained. The *RTD* resistance is measured by an excitation module, which provides a constant DC current of 100 μA and a sensing module, which measures the voltage across a *RTD*. The heater or *RTD* resistance is a function of its temperature. The resistance-temperature relation is obtained through a calibration process, which is achieved by placing the device in a temperature controlled oven and simultaneously recording the resistances and temperatures of the device. Typical resistance-temperature relations of heaters and *RTD*s are shown in Supplementary Fig. 5.

### Mass quality calculation

At critical composition, an equivalent mass quality, which estimates the fraction of initial mixture experienced phase separation, is defined as:

5 $${x}_{w}=\frac{{Q}_{tp}-{Q}_{sp}}{\dot{m}{\rm{\Delta }}{h}_{mix}({T}_{w})}$$

6 $${Q}_{sp}={h}_{sp}{A}_{heater}({T}_{w}-{T}_{f})$$

7 $${\rm{\Delta }}{h}_{mix}({T}_{w})=y{h}_{mix,l}+(1-y){h}_{mix,h}-{h}_{mix,critical}$$

where *Q*_*tp*_ is the two-phase flow total energy input to the fluid mixture, *Q*_*sp*_ is the single phase flow total energy input based on single phase heat transfer coefficient at the same temperature gradient as in the two-phase flow; *y* is the mass percentage of the lighter phase in the separated mixture of the two phases, and is calculated via the lever rule using the wall temperature; *Δh*_*mix*_ is the difference in heat of mixing according to the wall temperature.

### Flow visualization

The flow visualization videos are taken using a high-speed camera (phantom vision research v4.2) and a microscope (Leica TYPE020).

### The mixture preparation

The mixture of a certain concentration is prepared using mass scale of accuracy of 0.1 g.

## Electronic supplementary material

Supplementary Information

## Data Availability

The datasets generated during and/or analyzed during the current study are available from the corresponding author on reasonable request.
