# Supplementary material for: Advancing micro-scale cooling by utilizing liquid-liquid phase separation
Source: Sci Rep. 2018 Aug 14;8:12093. doi: 10.1038/s41598-018-30584-6 (PMC6092420; doi:10.1038/s41598-018-30584-6)
Supplement: Supplementary file 1 — Supplementary Information [file 41598_2018_30584_MOESM1_ESM.pdf]

Advancing micro-scale cooling by utilizing liquid-liquid phase separation

Wei Xing[1*], Amos Ullmann[2], Neima Brauner[2], Joel Plawsky[3], and Yoav Peles[4*]

[1]Department of Mechanical, Aerospace and Nuclear Engineering, Rensselaer Polytechnic Institute, 110 8th Street, Troy, NY 12180, USA
[2]School of Mechanical Engineering, Faculty of Engineering, Tel-Aviv University, Tel-Aviv, Israel
[3]Howard P. Isermann Department of Chemical and Biological Engineering, Rensselaer Polytechnic Institute, 110 8th Street, Troy, NY 12180, USA
[4]Department of Mechanical and Aerospace Engineering, University of Central Florida, 12760 Pegasus Blvd, Orlando, FL 32816, USA

Supplementary Information

Supplementary Figure 1

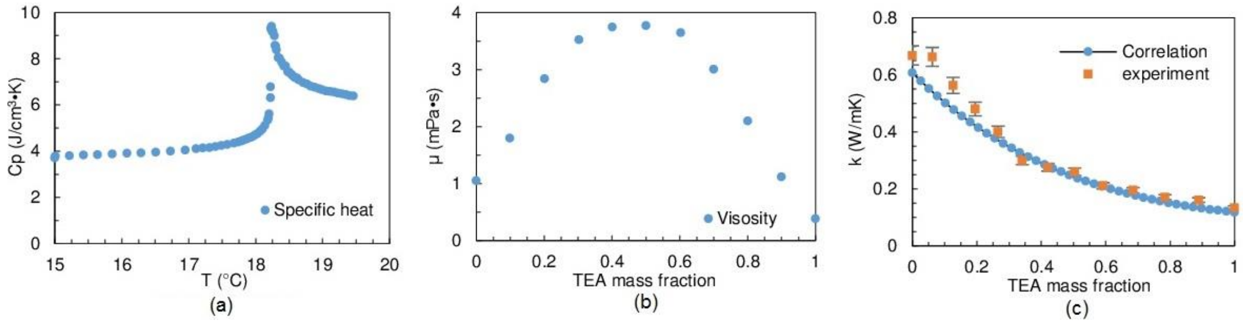

Figure S1. Physical/thermal properties of TEA/water system. (a) System's specific heat at critical composition[1]. (b) System's viscosity at 18 °C[2]. (c) System's thermal conductivity: correlation computed at 15 °C; experimental data taken at 15 °C. A correlation[3] is used to calculate the system's thermal conductivity due to the unavailability of experimental data. A simple validation experiment was performed using KD2-Pro to measure the system's thermal conductivity, and both data are in good agreement.

Supplementary Figure 2

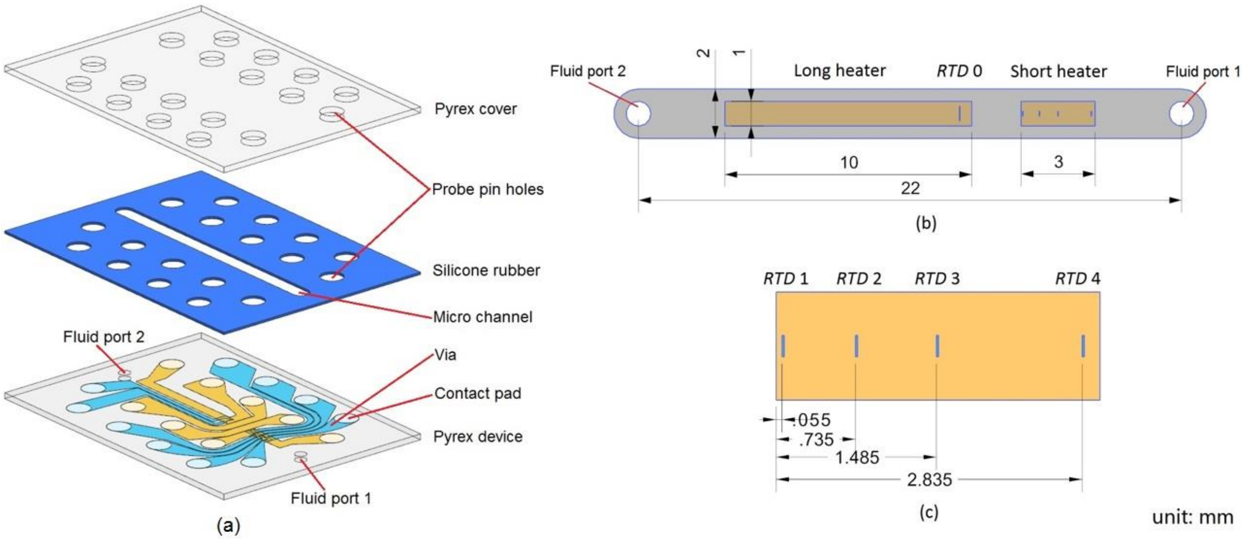

Figure S2. Micro fluidics device information. (a) Three-layer structure of the device. The fluid mixture flows from port 1 to port 2 during long heater average experiment; fluid flows from port 2 to port 1 during short heater average and local experiments. (b) Dimensions and configurations of the microchannel. The channel is 400 µm deep. (c) Locations of *RTD*s on the short heater. The Pyrex device is fabricated using standard clean room fabrication techniques, i.e. deposition, patterning and etching.

Supplementary Figure 3

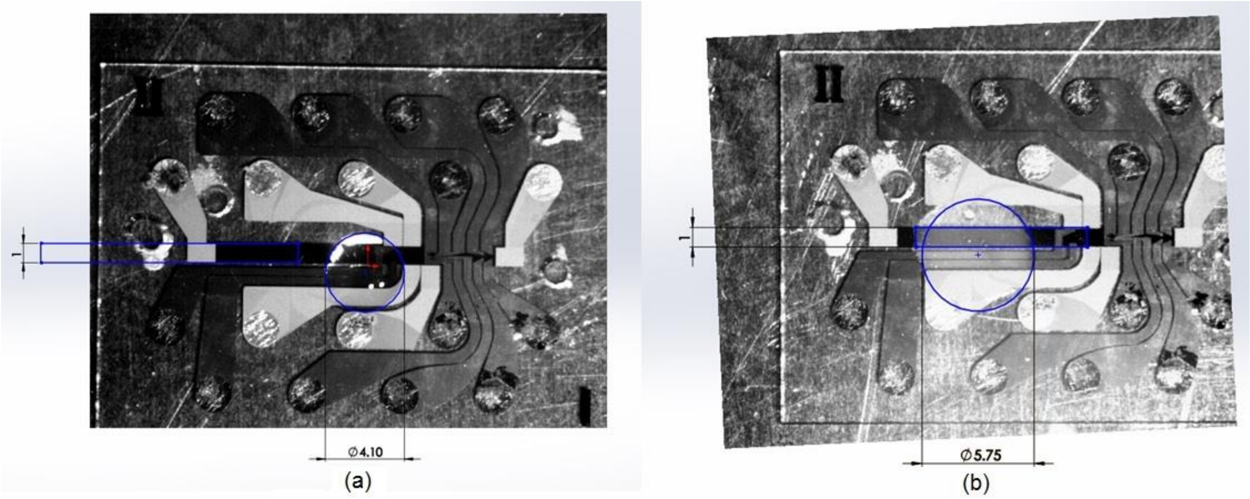

Figure S3. Water-rich phase and TEA-rich phase wettability comparison. (a) water-rich liquid; (b) TEA-rich liquid. Both droplets have the same volume (5 µL). Droplets were applied on the Pyrex device. The TEA-rich phase spread out and formed a larger circle than the water-rich phase, indicating greater wettability of the TEA-rich phase on the device. The heater width (1 mm) was used as a dimension indicator. Photos courtesy of Mr. Idan Shem Tov (Tel-Aviv University)

Supplementary Figure 4

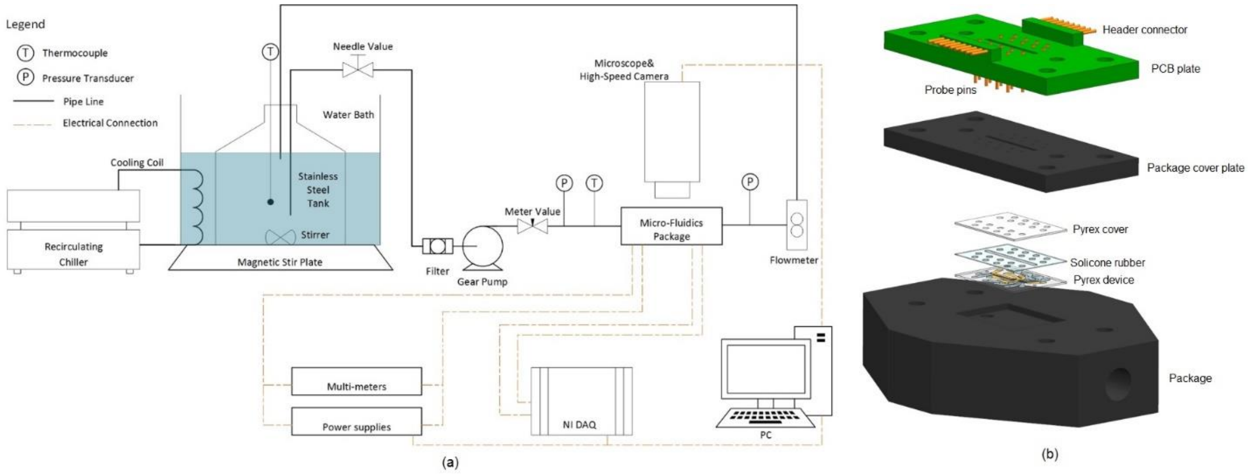

Figure S4. (a) Experimental loop; (b) Micro fluidics package. Header connectors and probe pins are soldered on the PCB plate to enable electrical connection with DC power supplies, multi-meters and National Instrument DAQ modules. The package cover plate provides mechanical pressure to the whole assembly and prevents fluid leakage.

Supplementary Figure 5

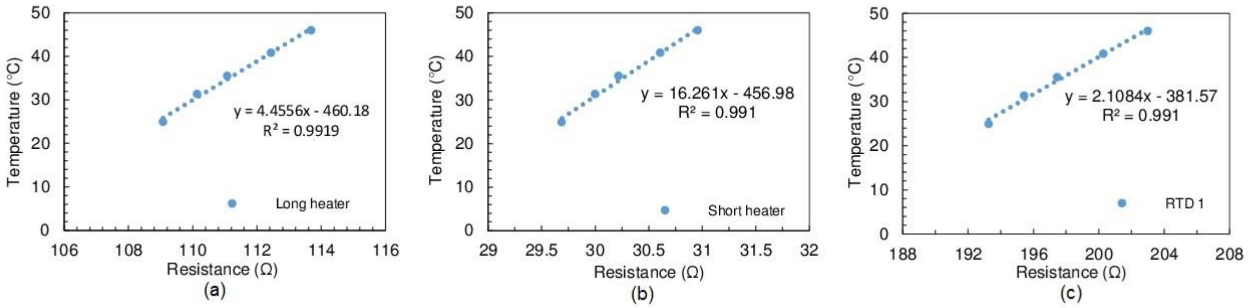

Figure S5. Resistance-temperature calibration curves. (a) Long heater calibration curve. (b) Short heater calibration curve. (c) *RTD* calibration curve. The purpose of calibrating heaters and *RTD*s is to obtain the relation between the heater/*RTD* resistance and temperature. The calibration process is carried by placing the micro fluidics device in a temperature controlled oven and measuring the resistances and the device temperature at the same time.

Supplementary Figure 6

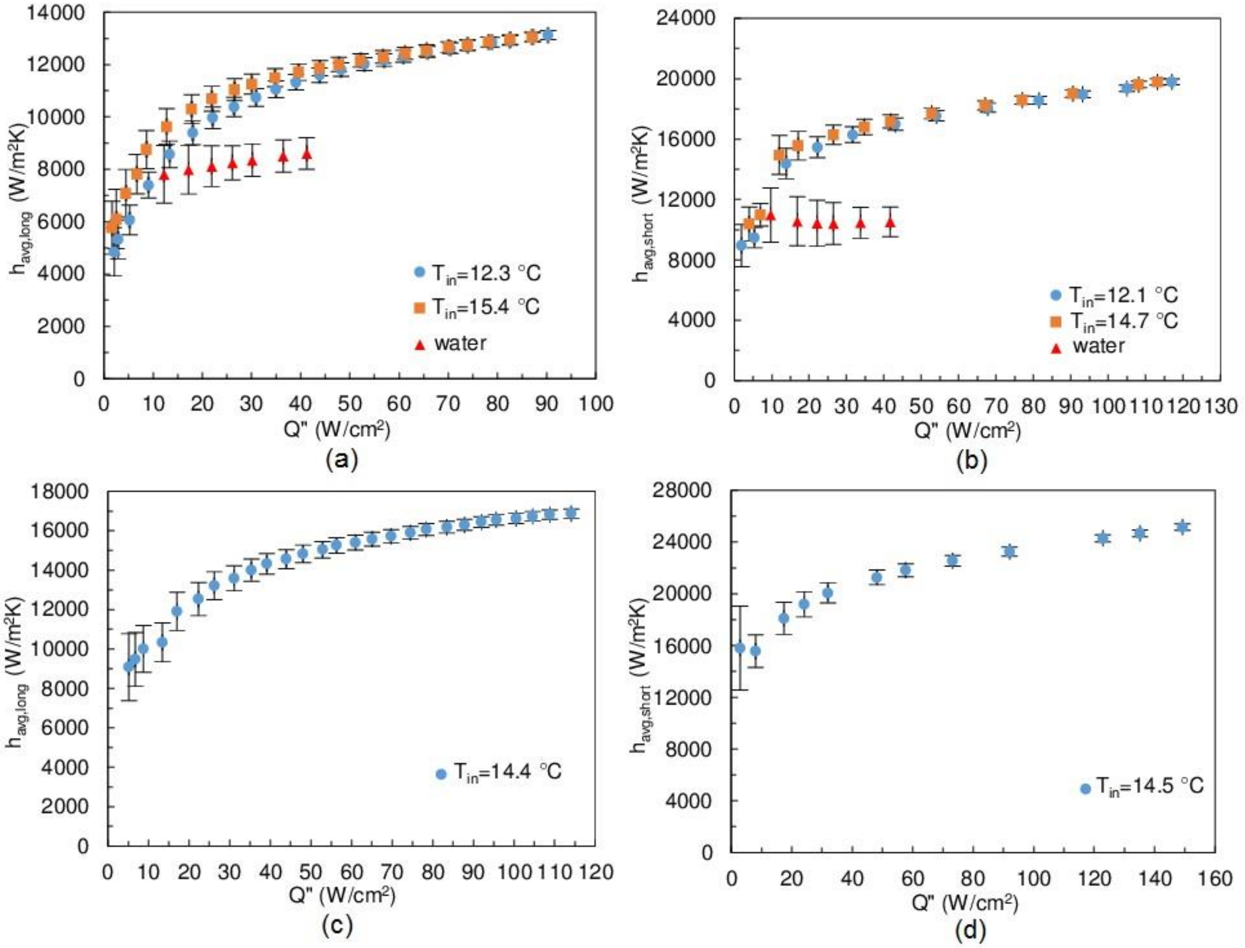

Figure S6. Critical Composition average heat transfer coefficient for long and short heater at various operating conditions. (a) long heater, m”=360 kg/m^{2}s, m”$_{water}$=375 kg/m^{2}s. (b) short heater, m”=398 kg/m^{2}s, m”$_{water}$=375 kg/m^{2}s. (c) long heater, m”=758 kg/m^{2}s. (d) short heater m”=758 kg/m^{2}s. Short heater has greater heat transfer coefficient due to the entry length effect.

Supplementary Figure 7

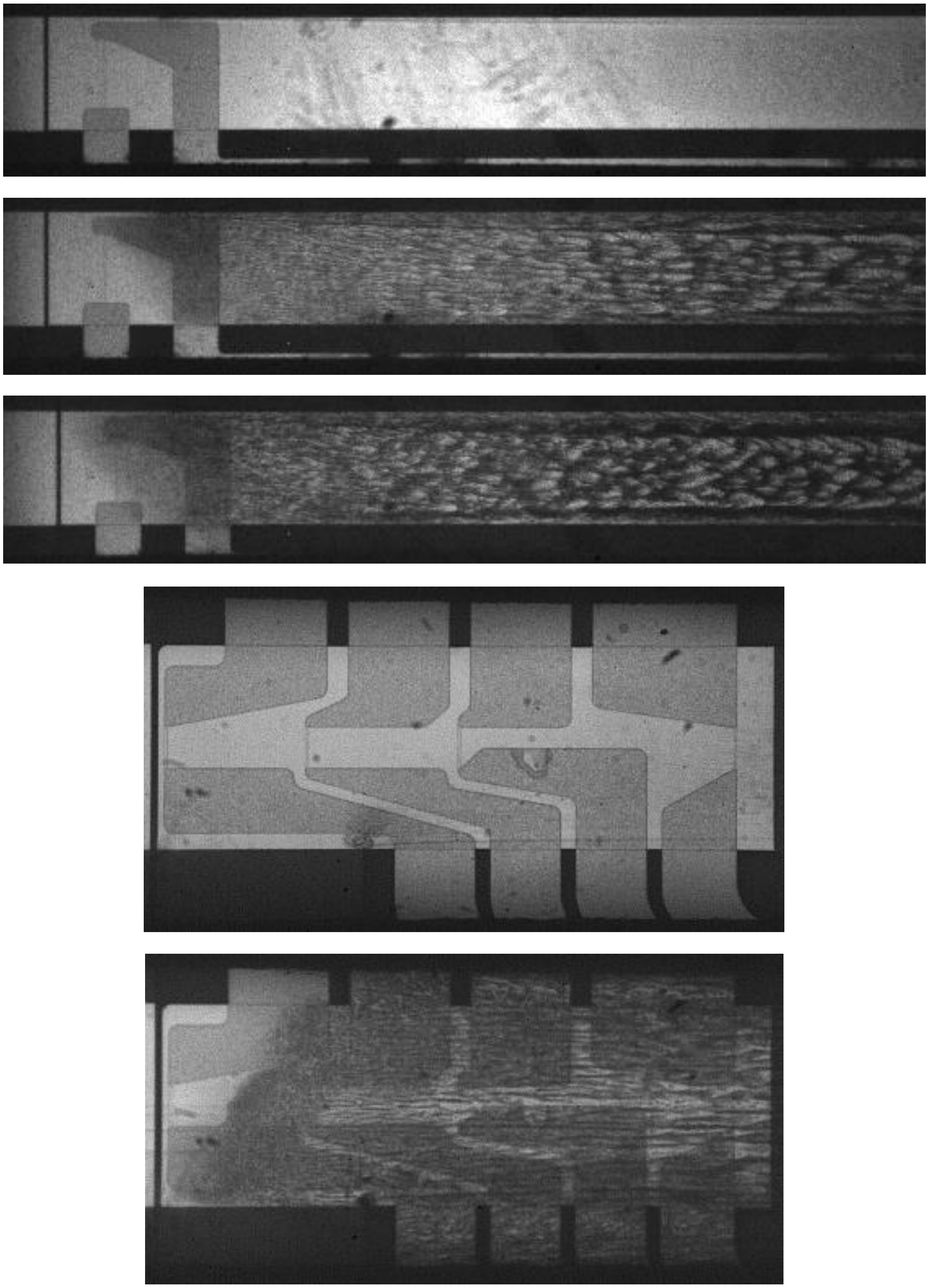

Figure S7. Full resolution flow visualization images for the critical composition mixture. Top 3 figures were taken on the long heater with m”=208 kg/m^{2}s, T_{in}=11.4 °C. Q”=4.0 W/cm^2, 17.8 W/cm^2, and 73.8 W/cm^2 from top to bottom. Bottom 2 figures were taken on the short heater with m”=284 kg/m^{2}s, T_{in}=14.4 °C. Q”=4.0 W/cm^2 and 52.8 W/cm^2, respectively.

Supplementary Figure 8

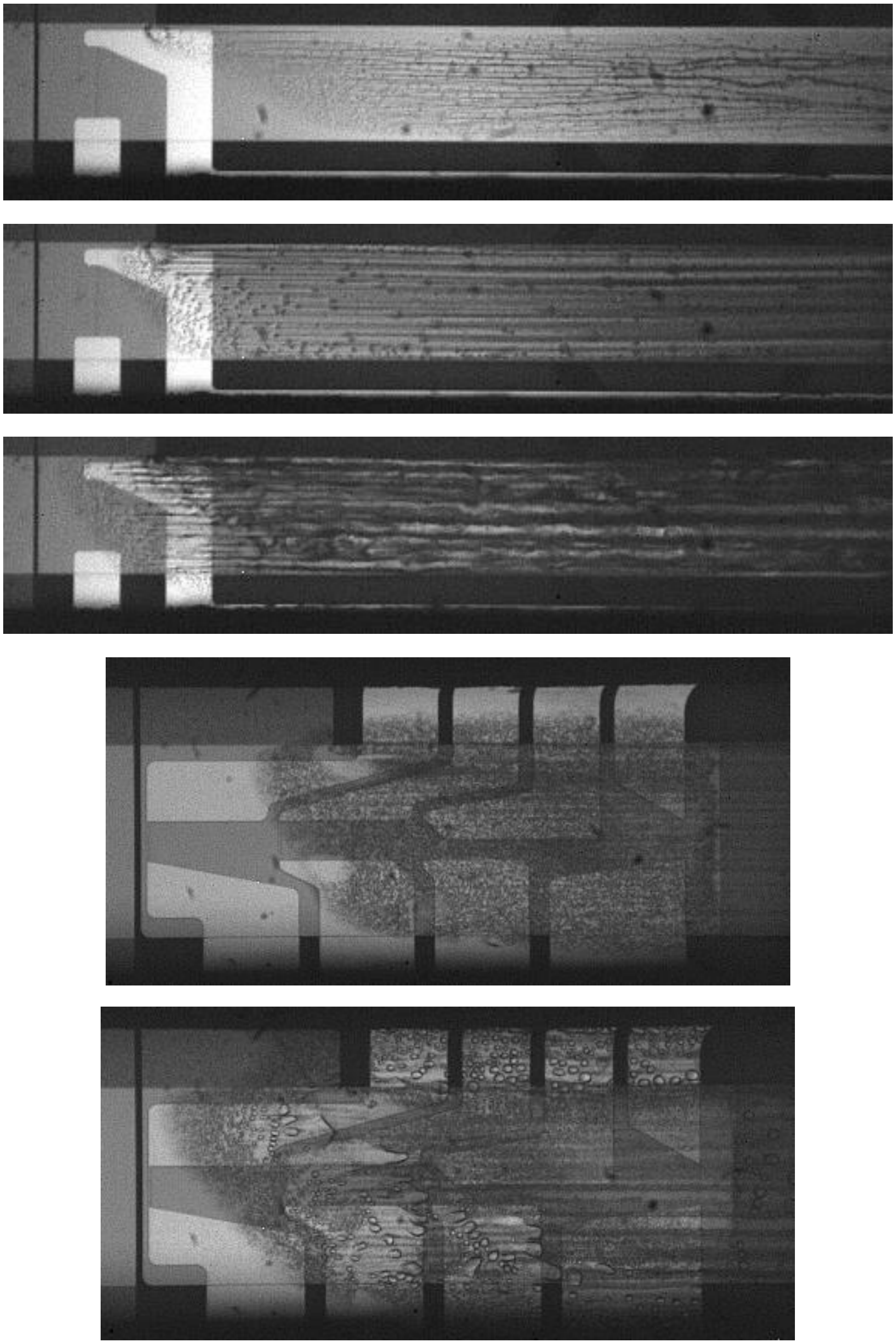

Figure S8. Full resolution flow visualization images for the 15% TEA mass fraction mixture. Top 3 figures were taken on the long heater with m"=241 kg/m^{2}s, T_{in}=12.8 °C. Q"=5.3 W/cm^2, 15.8 W/cm^2, and 47.0 W/cm^2 from top to bottom. Bottom 2 figures were taken on the short heater with m"=215 kg/m^{2}s, T_{in}=14.6 °C. Q"=4.0 W/cm^2 and 52.8 W/cm^2, respectively.

Supplementary Figure 9

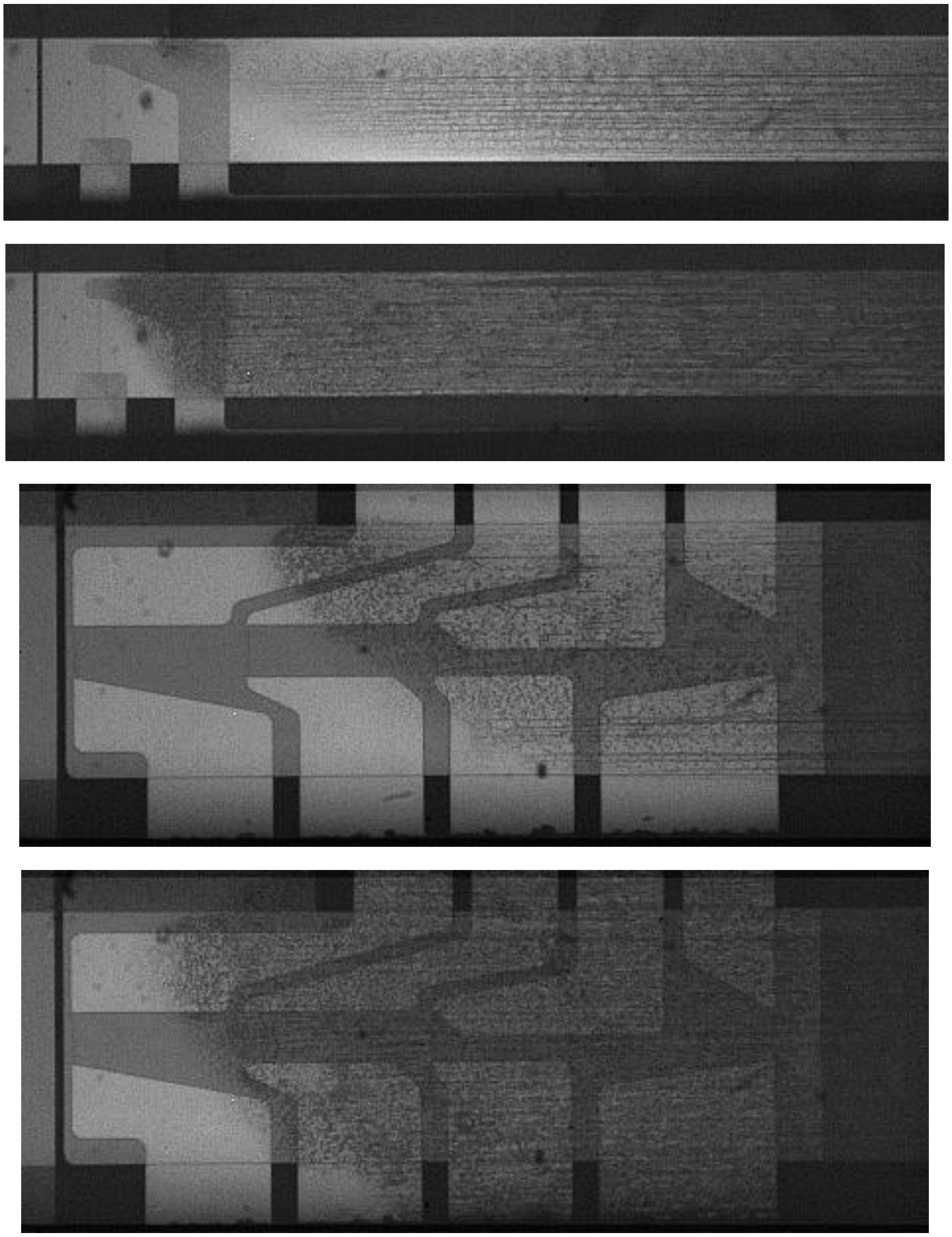

Figure S9. Full resolution flow visualization images for the 50% TEA mass fraction mixture. Top 2 figures were taken on the long heater with m''=178 kg/m^{2}s, T_{in}=12.2 °C. Q''=2.9 W/cm^2 and 10.2 W/cm^2, respectively. Bottom 2 figures were taken on the short heater with m''=358 kg/m^{2}s, T_{in}=14.5 °C. Q''=2.2 W/cm^2 and 14.8 W/cm^2, respectively.

Supplementary Note 1. Thermodynamics of solution behaviors.

Any fluid mixture system evolves toward the minimum Gibb's free energy[4,5]. The change of system's free energy is quantified by the Gibb's free energy of mixing, Δg_{mix}.

$$\Delta g_{mix} = g_{mixed} - g_{unmixed} = \Delta h_{mix} - T\Delta s_{mix} \quad (1)$$

When Δg_{mix} is less than zero, the mixed state has lower system free energy. Therefore, the mixed state, i.e., single phase mixture, is the stable state. On the contrary, when Δg_{mix} is greater than zero, the fluid will remain in the unmixed state as a multi-phase mixture.

For an ideal mixture, Δh_{mix} is assumed to be zero, i.e., no change in enthalpy state before and after mixing. For random mixing, the change in entropy is expressed as

$$\Delta s_{mix} = -R(x_A ln x_A + x_B ln x_B) \quad (2)$$

Where, x_A and x_B are the mole fractions of component A and B, respectively, and R is the universal gas constant. Note that x_A and x_B are values between 0 and 1, such that ln(x_A) and ln(x_B) are negative and thus Δs_{mix} is greater than zero. This leads to

$$\Delta g_{mix} = g_{mixed} - g_{unmixed} = -T\Delta s_{mix} < 0 \quad (3)$$

Thus, an ideal mixture is always miscible at any concentration and temperature.

However, the ideal mixture assumption is not always true. One deviation is that Δh_{mix} is not always zero. Δh_{mix} represents the difference of bonding energy between molecules of different species and of the same species, assuming random mixing.

$$\Delta h_{mix} = \frac{z}{2} x_A x_B (2\omega_{AB} - \omega_{AA} - \omega_{BB}) = \frac{z}{2} x_A x_B \omega \quad (4)$$

Where,

z is the number of bonds between molecules,

ω_{AB} is the energy of A-B molecule interaction,

ω_{AA} is the energy of A-A molecule interaction,

ω_{BB} is the energy of B-B molecule interaction,

ω is the bond energy difference between A-A interaction and B-B interaction.

Assuming the above forms of Δh_{mix} and Δs_{mi} yields the thermodynamics model of regular solution.

$$\Delta g_{mix} = \frac{z}{2} x_A x_B \omega - T[-R(x_A ln x_A + x_B ln x_B)] \quad (5)$$

When ω<0, the system favors mixing, i.e., A-B interaction is weaker than the average of A-A and B-B interaction. However, the situation becomes complicated as ω>0, and the temperature determines the sign of Δg_{mix}. It can be seen from Fig. N1 that as ω>0, the system is completely miscible at high temperatures, and is partially miscible at low temperatures. Thus, the phase separation is brought by reducing the mixture temperature. Such a system is an Upper Critical Solution Temperature (UCST) system. Note that the system is always miscible at extreme compositions, i.e., very close to 0 or 1. Real solution behavior deviates from the regular solution model, and needs to be analyzed case by case.

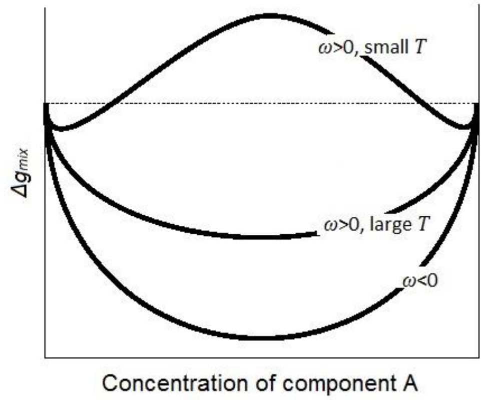

Figure N1. Gibb's free energy of mixing of a binary system.

Supplementary Note 2. Thermodynamics consideration of triethylamine (TEA)/water mixture

TEA/water mixture is very special due to its Lower Critical Solution Temperature (LCST) behavior. When the system is chilled below its critical temperature and remains as a single phase mixture, hydrogen bonds form between the amine part of TEA and water molecules. Thus, the TEA-water interaction is stronger than the TEA-TEA interaction and water-water interaction, indicating a positive ω. The system needs to absorb thermal energy to break the hydrogen bonds during phase separation. Moreover, the presence of hydrogen bonds changes the entropy behavior of the system. The system processes lower entropy in the mixed state than the unmixed state, since the hydrogen bonds organize the molecules. Thus, the single phase mixed state is less chaotic than the unmixed states. As a result, the Δs_{mix} term is less than zero. This unfavorable behavior of entropy causes the system to undergo phase separation with increased temperature.

Supplementary Note 3. Nucleation and spinodal decomposition

Nucleation and spinodal decomposition are two mechanisms involved in phase separation process[6,7]. Fig. N2 illustrates the relation between system's free energy and phase separation mechanisms. The top figure represents a typical twin-well shape binary system Gibb's free energy at a specific temperature. In Region I, the system starts with 100% component A. When adding small amount of component B, the system moves toward a lower free energy state, thus the system is miscible until reaching composition x_1. In Region II, when adding component B, the system's free energy increases. This does not meet the global criterion for system stability. However, the Gibb's free energy curve remains concave, such that the system satisfies the local stability criterion, i.e., locally, the system's free energy after mixing is less than the free energy before mixing. As a result, Region II (space between x_1 and x_2) corresponds to a meta-stable state. If a system stays in a meta-stable state, phase separation is by means of nucleation. On the phase diagram, Region II corresponds to the gap between the binary curve and spinodal curve. For this process, an energy barrier needs to be overcome in order to trigger the phase transition process. As more B species is added, the curvature of the free energy changes and becomes convex (Region III). In this region, both global and local stability criteria are not satisfied. Consequently, the system is in an unstable state. Phase separation takes place by means of spinodal decomposition, which is an energy relaxation process with no energy barrier to overcome.

The nucleation process features with localized growth of spherical domains and needs to be triggered with substantial perturbations. Spinodal decomposition is a more intensive process with rapid domain growth and coalescence. For a UCST/LCST system at its critical composition, spinodal decomposition happens at wherever the mixture temperature is below/over the critical value. For quiescent fluid systems, spinodal composition is recognized by bi-continuous and dendritic domains. More detailed studies on spinodal decomposition further treat the process in two stages, diffusion and convection. The domain growth rate in the diffusion stage is proportional to $t^{1/3}$ (t is time), and linear with t in the convection stage. Under strong bulk convection and shear force, the domain morphology of spinodal decomposition is shown to be elongated droplets or string-shape.

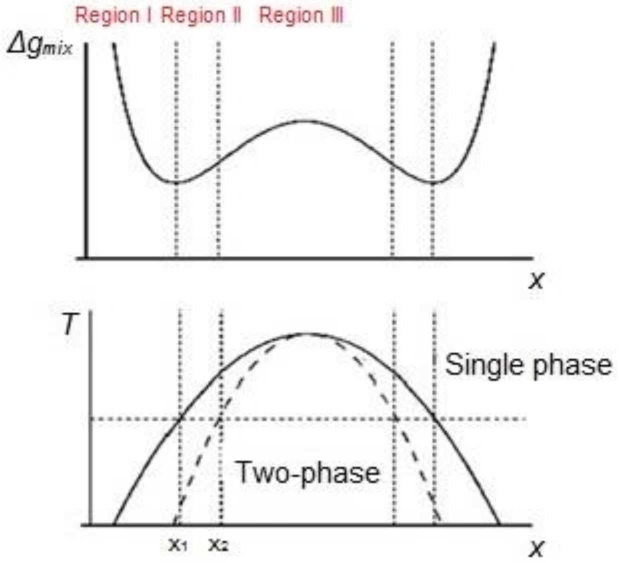

Figure N2. Illustration of Gibb's free energy and phase diagram. Top figure: typical twin-well potential; bottom figure: phase diagram for a UCST system; solid line is binary curve, dashed line is spinodal curve.

Supplementary Note 4. Single phase flow conjugate heat transfer model for estimating the separation layer thickness.

In order to estimate the separation boundary layer thickness, a conjugate heat transfer model is established. The model simulates the heat transfer in both fluid flow and device substrate. A hypothetical single phase fluid is incorporated into the model. The hypothetical fluid has the same properties as TEA/water mixture at its critical composition and at a temperature just below the critical temperature, but with a varying specific heat as shown Fig. S1(a). Such a configuration is able to simulate the effect of heat of mixing on the heat transfer and isolate the boost caused by the flow mixing. The simulation is done by COMSOL Multiphysics®, and the meshing size has been optimized to ensure accuracy and efficient computing. The comparison of average wall temperature between the experiment and simulation is shown in Fig. N4(a). At low heat fluxes, the flow remains as single phase flow, and our simulation yields good agreement with the experimental data. However, as the applied heat flux increases, the fluid mixture experiences phase transition and provides enhanced thermal transport. As a result, the actual average wall temperature is significantly lower than the single phase model prediction. There are two major reasons that account for the deviation, first, simulation cannot capture the flow mixing effect; and second, the change in physical/thermal properties after phase separation is not taken into consideration.

To overcome the abovementioned issues, an effective thermal conductivity, k_{eff}, is proposed. The k_{eff} is applied to the fluids within the separation boundary layer. This is realized by setting the mixture thermal conductivity as a function of temperature. When T<18.2 °C, the mixture k is used in the model; when T≥18.2 °C, k_{eff} is active in the model. The value of k_{eff} is set such that the simulation average wall temperature is as close as possible to the experimental value. It is possible that the value of k_{eff} is dependent upon the flowrate, residence time, heater length and heat flux (or fluid temperature). For simplicity, k_{eff} is found by trial and error with the least sum of square difference with the experimental wall temperature. The k_{eff} is found to be 0.68 W/(mK), and using k_{eff} leads to good agreement between the simulation and experimental wall temperatures at various flow conditions (Fig. N4(b)-(e)). The separation layer thickness (δ_s) is then predicted using the conjugate heat transfer model with both temperature dependent specific heat and thermal conductivity.

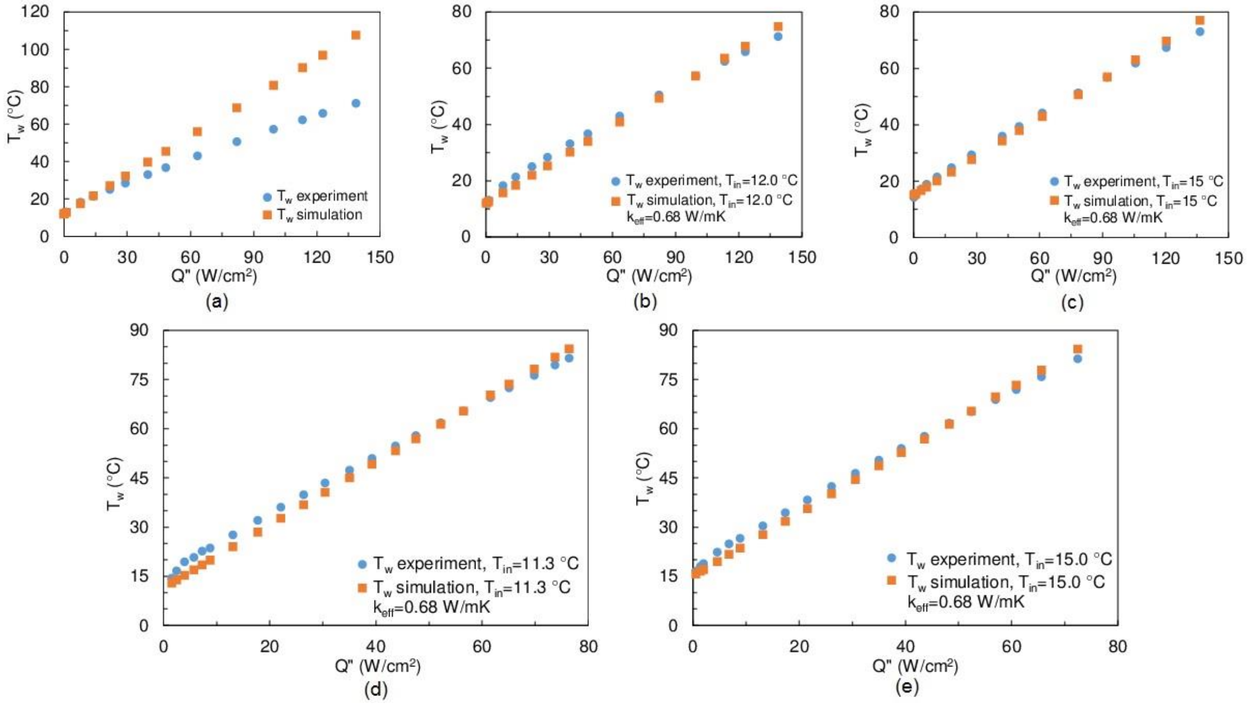

Figure. N2 Comparison between single phase conjugate heat transfer model wall temperature prediction and experimental wall temperature measurement. Blue circle represents experimental value, and orange square represents simulation values. (a) short heater, m"=567 kg/m^{2}s, T_{in}=12.0 °C. (b) short heater, m"=567 kg/m^{2}s, T_{in}=12.0 °C. (c) short heater, m"=567 kg/m^{2}s, T_{in}=15.0 °C. (d) long heater, m"=208 kg/m^{2}s, T_{in}=11.3 °C, (e) long heater, m"=208 kg/m^{2}s, T_{in}=15.0 °C. The hypothetic fluid has the exact same specific heat of the mixture in all figures, an effective thermal conductivity k_{eff}=0.68 W/mK is used in figure (b), (c), (d) and (e).

References

1. Flewelling, A. C., DeFonseka, R. J., Khaleeli, N., Partee, J. & Jacobs, D. T. Heat capacity anomaly near the lower critical point of triethylamine-water. *J. Chem. Phys.* **104,** 8048–8057 (1996).
2. Hafaiedh, N. *et al.* Dynamic Viscosity Study of Binary Mixtures Triethylamine + Water at Temperatures Ranging from (283 . 15 to 291 . 35) K. 2195–2199 (2009).
3. Li, C. C. Thermal conductivity of liquid mixtures. *AlChe J.* **11,** 927–930 (1976).
4. Walas, S. *Phase Equilibria in Chemical Engineering*. (1985).
5. Atkins, P. & DePaula, J. *Physical Chemistry*. (1985).
6. K. Kubota, N. K. Spinodal Decomposition in a Binary Mixture. *Phys. Rev. Lett.* **68,** 197–200 (1992).
7. Poesio, P., Cominardi, G., Lezzi, a., Mauri, R. & Beretta, G. Effects of quenching rate and viscosity on spinodal

decomposition. *Phys. Rev. E* **74,** 11507 (2006).
